# Non-Invasive Assessment of Intravascular Pressure Gradients: A Review of Current and Proposed Novel Methods

**DOI:** 10.3390/diagnostics9010005

**Published:** 2018-12-29

**Authors:** Tin-Quoc Nguyen, Kristoffer Lindskov Hansen, Thor Bechsgaard, Lars Lönn, Jørgen Arendt Jensen, Michael Bachmann Nielsen

**Affiliations:** 1 Department of Diagnostic Radiology, Copenhagen University Hospital, Blegdamsvej 9, 2100 Copenhagen, Denmark; lindskov@gmail.com (K.L.H.); lonn.lars@gmail.com (L.L.); mbn@dadlnet.dk (M.B.N.); 2 Department of Clinical Medicine, University of Copenhagen, Blegdamsvej 3B, 2200 Copenhagen, Denmark; 3 Department of Radiology, Odense University Hospital Svendborg Hospital, Baagøes Alle 31, 5700 Svendborg, Denmark; thorbechsgaard@gmail.com; 4 Center for Fast Ultrasound Imaging, DTU Elektro, Technical University of Denmark, Ørsteds Plads Building 349, 2800 Lyngby, Denmark; jaj@elektro.dtu.dk

**Keywords:** pressure gradient, ultrasound, magnetic resonance imaging, intravascular catheterization, review

## Abstract

Invasive catheterization is associated with a low risk of serious complications. However, although it is the gold standard for measuring pressure gradients, it induces changes to blood flow and requires significant resources. Therefore, non-invasive alternatives are urgently needed. Pressure gradients are routinely estimated non-invasively in clinical settings using ultrasound and calculated with the simplified Bernoulli equation, a method with several limitations. A PubMed literature search on validation of non-invasive techniques was conducted, and studies were included if non-invasively estimated pressure gradients were compared with invasively measured pressure gradients in vivo. Pressure gradients were mainly estimated from velocities obtained with Doppler ultrasound or magnetic resonance imaging. Most studies used the simplified Bernoulli equation, but more recent studies have employed the expanded Bernoulli and Navier–Stokes equations. Overall, the studies reported good correlation between non-invasive estimation of pressure gradients and catheterization. Despite having strong correlations, several studies reported the non-invasive techniques to either overestimate or underestimate the invasive measurements, thus questioning the accuracy of the non-invasive methods. In conclusion, more advanced imaging techniques may be needed to overcome the shortcomings of current methods.

## 1. Introduction

Intravascular pressure gradients are important to monitor to understand the cardiovascular system. High gradients across stenotic heart valves may cause symptoms and disability, or even death [1]. The gold standard is an invasive procedure involving fluoroscopy for guidance, but non-invasive cost-effective alternative techniques are needed [2,3,4,5]. Vascular access for diagnostic and therapeutic procedures can be performed through various vessels with the femoral artery, radial artery, and jugular vein being the most widely used access sites [6,7]. Two main pathways exist for invasive measurements: fluid-filled catheters that transmit pressure-waves to an external pressure sensitive transducer [8,9], or directly on the pressure sensitive tip of a pressure wire [9].

Invasive catheterization is associated with various complications, such as bloodstream infections [10], neurological deficits [11], hematomas and pneumothorax [6]. Additionally, intravascular tools may narrow the vessel lumen, and ultimately interfere with the measurements [9,12,13]. Catheterization is considered unsuitable for regular follow-up [14] and is not recommended for routine diagnostic pressure assessment [15,16].

Alternatives to invasive methods are pressure gradients obtained with imaging, such as ultrasound (US) and magnetic resonance imaging (MRI). By using different equations, acquired velocity estimations can be converted to pressure gradients [17,18,19,20,21]. The aim of this paper was to review in vivo studies of non-invasively derived pressure gradients compared with invasive methods to assess the performance of non-invasive pressure gradient estimation in the literature.

## 2. Literature Search

A literature search on non-invasive techniques was performed in PubMed on 19 December, 2018, using the following search criteria: ((“Pressure”[Mesh] OR “Blood Pressure”[Mesh] OR “Blood Pressure Determination”[Mesh] OR “Arterial Pressure”[Mesh]) AND “Diagnostic Imaging”[Mesh]) AND (“Catheterization” OR “Catheterisation”). This resulted in 3978 papers that were narrowed down to 338 papers by adding the following search criteria: (“Pressure gradient” OR “Pressure drop” OR “Pressure difference”).

The papers were screened by title and abstract. Inclusion criteria were English language and comparisons of non-invasive pressure gradient estimation versus invasive methods, in vivo. Full-text reading was done on the selected papers and additional relevant studies found from the reference list of these papers were included. In total, 38 publications were included in this review. 

## 3. From Images to Pressure Gradients

Blood accelerates when passing through a stenosis [17]. The conversion of potential energy to kinetic energy results in a high flow velocity and a drop in pressure [22]. As the diameter widens distal to a stenosis, flow decelerates and pressure rises again, albeit to a lower pressure level than initially, as some kinetic energy is removed due to viscous losses and the formation of turbulences [22]. Pressure gradients cannot be measured directly with imaging modalities. Yet, velocities can be obtained and used to calculate pressure gradients. The most commonly used formulas to calculate pressure gradients from velocities are the simplified Bernoulli equation and the Navier-Stokes equations.

The Bernoulli equation is derived from the principle of conservation of energy [18] and is used to convert velocities to pressure gradients. By assuming peak velocities are acquired, that a large difference in proximal and distal velocity is present, and by neglecting viscous losses, the equation is expressed by the simplified Bernoulli equation as

(1) $$\Delta p=4\;\times \;v_{2}{}^{2}$$

where Δp is the pressure gradient and $v^{2}$ is the measured peak velocity in the stenosis [17,18]. The simplified Bernoulli equation has several shortcomings due to the assumptions made. Firstly, it cannot be used if the distal velocity approaches the proximal velocity. Secondly, it is inaccurate for narrow and long stenoses, where viscous losses are significant [18]. Thirdly, flow is assumed to be laminar in one direction with a constant velocity, and thus neglects the complexity of hemodynamics [23]. Additionally, the equation may result in overestimation of the invasively measured pressure gradient, if the downstream invasive measurement is not performed close to the stenosis [18]. 

If the distal velocity approaches the proximal velocity, the proximal velocity can no longer be neglected, and $v^{2}$ has to be replaced with the distal and proximal velocities. This can be expressed by the expanded Bernoulli equation as

(2) $$\Delta p=4\times \left(v_{2}{}^{2}-v_{1}{}^{2}\right)$$

where $v^{2}$ is the distal flow velocity, and $v^{1}$ the proximal velocity [18].

The Navier–Stokes equations describe flow hemodynamics more accurately than the Bernoulli equation [23] and can be used to calculate pressure gradients from velocity fields. Fluids are assumed to be incompressible and Newtonian [24]. The equations are derived from the laws of mass conservation and linear momentum [19,20,21] and can be expressed as

(3) $$-\nabla p=\rho \left(\frac{\partial v}{\partial t}+v\times \nabla v-g\right)-\mu \nabla^{2}v$$

where *p* is pressure, ρ is fluid density, ∂v/∂t is temporal acceleration, ∇ is divergence, v is velocity, μ is fluid viscosity, and g is the gravitational force. The Bernoulli equation is a simplification of the Navier–Stokes equations [23], as it neglects the temporal acceleration, the viscous losses and the gravitational forces. The Navier–Stokes equations do not make these assumptions, but require an increased amount of measurements of the full velocity field for estimating pressure gradients [25]. 

## 4. Imaging Modalities

Both the Bernoulli and the Navier–Stokes equations depend on flow velocities being measured to calculate pressure gradients across stenoses. Blood flow velocities can be obtained non-invasively with different medical imaging techniques, i.e., MRI and US.

### 4.1. Magnetic Resonance Imaging

MRI is a non-invasive imaging modality depicting detailed anatomical structures without using ionizing radiation [26,27]. MRI utilizes the changes in hydrogen-nuclei being exposed to radiofrequency signals inside a strong magnetic field.

**Figure 1:**
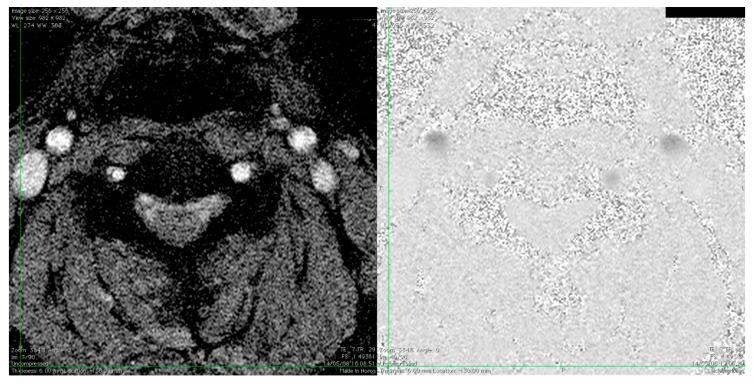
The **left** image shows a time-of-flight image of the neck in the transverse scan plane. The image is used for identifying and marking the carotid arteries for velocity estimation with phase contrast MRI. The **right** image shows the phase contrast image using a through-plane sequence. Images were provided by co-author Kristoffer Lindskov Hansen.

Flow velocities can be obtained with Phase Contrast MRI (PC-MRI), which measures the phase shift of moving spins [28]. The net phase shift is proportional to the flow velocity (Figure 1) [29,30].

MRI is widely considered the gold standard for estimation of flow velocity and volume flow [31,32,33,34]. MRI is known to have high spatial resolution (approximately 100 µm) and good contrast for tissue differentiation, but drawbacks are that MRI is expensive, relatively time consuming and can be difficult to perform on patients with certain metallic foreign bodies or claustrophobia [35,36,37]. Additionally, conventional cardiac PC-MRI is gated with electro-cardiogram and has to be performed over multiple cardiac cycles, but alternative MRI-techniques are emerging, allowing for real-time velocity estimation [38].

### 4.2. Ultrasound

US is a non-invasive imaging modality capable of producing real-time visualization of anatomical structures without adverse effects [39]. Sound waves are emitted from the US transducer, and the strength of the sound waves reflected back depends on the tissue medium they have interacted with. Flow velocities can be obtained with Doppler US by examining the change in frequency of the returning sound wave. The movement of red blood cells in flowing blood increase or decrease the frequency of emitted sound waves depending on their flow direction, and the measured blood velocity is proportional to the returning shift in Doppler frequency [40].

**Figure 2:**
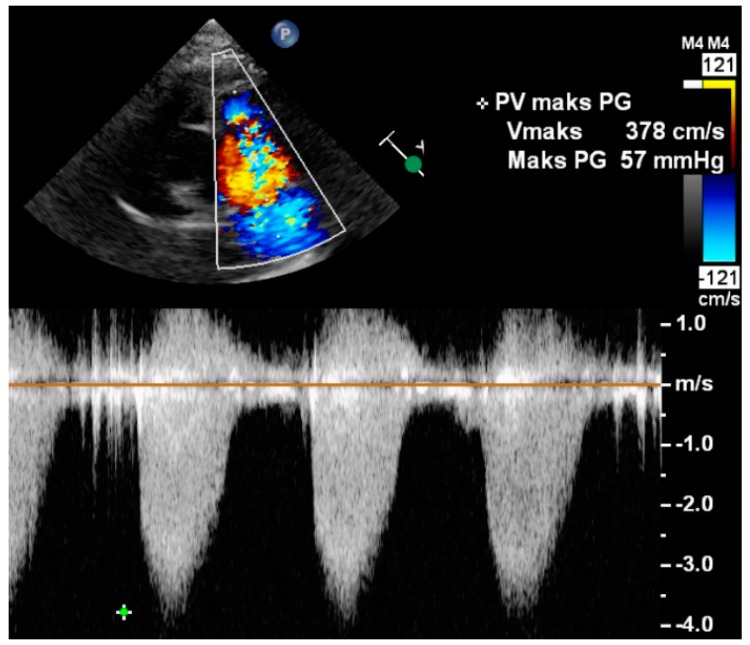
Continuous wave Doppler ultrasound of flow across the pulmonary valve in a patient with pulmonary valve stenosis. Peak pressure gradient (“Maks PG”) shown in upper right corner of the screen was calculated in real-time from peak velocity by using the simplified Bernoulli equation. Image was provided by Klaus Juul (Department of Pediatric Cardiology, Copenhagen University Hospital, Denmark).

US is a portable, real-time diagnostic imaging modality with low expenses, with high spatial resolution (2.5 µm), and high temporal resolution [39,41]. Limitations include a small field of view, angle dependent velocity estimation and suboptimal insonation windows [15,42,43,44]. Guidelines recommend using continuous wave Doppler US for measuring peak velocities and peak pressure gradients across aortic and tricuspid valves [15,45], as the technique allows for evaluation of higher velocities than pulsed wave Doppler (Figure 2). However, a disadvantage of using continuous wave Doppler US is that no information of depth is obtained, as the acquired peak velocity can be from anywhere along the interrogation line [46].

## 5. Estimation of Pressure Gradients in the Heart and the Larger Thoracic Arteries

The recommended initial imaging modality for diagnosing diseases in the cardiovascular system is transthoracic echocardiography [16,45,47], though MRI is also widely used for imaging of the chest [48,49]. In this section, results from studies of the cardiovascular system evaluating the accuracy of estimated pressure gradients compared with invasively measured pressure gradients will be reviewed.

### 5.1. Aortic and Mitral Valves

Valve area is included in the reference measurement for assessing aortic valve stenosis severity and estimated with transthoracic echocardiography using planimetry [16]. However, the severity of a stenosis cannot be evaluated from this measurement alone. Other parameters like the transvalvular peak velocity and peak pressure gradient must also be considered. The transvalvular estimated pressure gradient is a robust measure and is used as a prognostic indicator [22,50]. Several studies have evaluated the accuracy of transvalvular estimated pressure gradient derived from Doppler US using the simplified Bernoulli equation (Equation (1)) by comparing with an invasively measured pressure gradient. Studies conducted between the late 1970s and 1980s reported strong correlations between estimated and invasively measured pressure gradients for both aortic and mitral valve stenosis (*r* = 0.72 to 0.97) [17,42,46,51,52,53,54,55,56], with the majority reporting the estimated pressure gradients to underestimate the invasively measured ones [17,42,46,51,52]. Inaccurate angling between Doppler beam and peak blood flow velocity was suggested as the main reason for this. Hatle et al. found the underestimation to be more pronounced in patients >50 years old and suggested that anatomical changes, such as valve deformity and calcifications, could induce a greater variation in peak flow direction [52].

Contrarily, other studies reported the estimated pressure gradients to overestimate the invasively measured values (mean bias: +0.4 to +19 mmHg) [57,58,59]. Overestimation by Doppler US was suggested to be caused by a phenomenon called “pressure recovery” [57,58], by which the discrepancy between the methods is caused by improper placement of the catheter during measurement of the distal intravascular pressure [18]. The distal pressure should ideally be measured with the catheter placed immediately downstream of the stenosis, as the increase in pressure does not occur immediately after passing a stenosis, but will instead happen as a steady increase downstream [58].

Baumgartner et al. suggested a method to correct for pressure recovery and reported both “non-corrected estimated pressure gradients” and “corrected estimated pressure gradients” to correlate strongly with invasively measured pressure gradients (*n* = 21; *r* = 0.93 to 0.97) [58]. The authors reported non-corrected estimated pressure gradients to significantly overestimate the invasively measured ones (mean bias: +18 mmHg), while the corrected estimated pressure gradients did not (mean bias: +0.4 mmHg). However, when Yamazawa et al. applied the suggested correction to their velocity data obtained with Doppler US, mean bias for estimated pressure gradients changed from overestimating, when non-corrected, to instead underestimating the invasive measurements, when corrected (*r* = 0.87 to 0.79, mean bias: +8.5 mmHg to −9.1 mmHg) [60]. Eichenberger et al. examined 10 patients with aortic valve stenosis and three healthy subjects using Doppler US, PC-MRI and cardiac catheterization, and found estimated pressure gradients from both imaging modalities to correlate strongly with invasively measured pressure gradients (*n* = 13, Doppler US: *r* = 0.96; PC-MRI: *r =* 0.97) [61]. The pressure gradients were calculated by applying the simplified Bernoulli equation (Equation (1)) to estimated peak velocities.

### 5.2. Pulmonary Arteries

Patients suspected of pulmonary arterial hypertension are examined with transthoracic echocardiography to estimate the systolic pressure in the pulmonary artery, which is achieved by summating the pressure gradient across the tricuspid valve with the right atrial pressure [45]. In echocardiography, the tricuspid pressure gradient is obtained by applying the simplified Bernoulli equation (Equation (1)) to the tricuspid regurgitation velocity [45,62]. Hioka et al. reported estimated pressure gradients derived from Doppler US across the tricuspid valve to correlate well with invasively measured pressure gradients with a small mean bias (*n* = 55, *r* = 0.73, *p* < 0.001; mean bias: +2.5 mmHg) [63]. The bias became more pronounced the more severe the stenosis was [63]. Right atrial pressure is approximated by measuring the respiratory variation of the diameter of the inferior vena cava during a sniff maneuver. However, estimation of right atrial pressure by visually evaluating the inferior vena cava has limited precision [64,65]. Magnino et al. reported the average accuracy between non-invasive estimation of right atrial pressure and invasive pressures measured during right heart catheterization to be 34% [65].

The accuracy of echocardiography for pulmonary hypertension diagnosis was evaluated in a meta-analysis by Janda et al. [66]. The authors reported a moderate correlation between echocardiography and invasive catheterization (summary correlation coefficient: 0.70). The meta-analysis included 29 studies that compared pulmonary arterial hypertension assessed with Doppler US with the invasively measured pressures of right heart catheterization in 1998 patients. However, 41% of the patients had suboptimal tricuspid regurgitation velocities, which could potentially result in underestimation of the tricuspid pressure gradient. Because of the limitations, it was concluded that echocardiography should not be recommended as a stand-alone modality for pulmonary hypertension diagnosis.

Nogami et al. used PC-MRI to estimate pressure gradients across the tricuspid valve in 20 patients by applying the simplified Bernoulli equation (Equation (1)) to peak regurgitant velocities [67]. The estimated pulmonary artery systolic pressure strongly correlated with values obtained during right heart catheterization (*n* = 20, *r* = 0.94, *p* < 0.0001).

### 5.3. Coarctation of the Aorta

Transthoracic echocardiography, cardiac MRI, and cardiac computed tomography (CT) are the recommended methods for diagnosing patients suspected of coarctation, while cardiac catheterization is reserved for intravascular treatment [47]. Intervention is indicated if invasive peak-to-peak pressure gradient is measured to be greater than 20 mmHg, but may be less than 20 mmHg if radiological evidence of coarctation is significant. Studies evaluating the accuracy of estimated pressure gradients have shown varying results. Strong correlations were reported between Doppler US derived pressure gradients calculated with the expanded Bernoulli equation (Equation (2)) and pressure gradients measured with invasive catheterization (*n* = 32; *r* = 0.98 and *n* = 28; *r* = 0.76) [8,68]. When the simplified Bernoulli equation (Equation (1)) was used, correlations remained strong (*r* = 0.91 and *r* = 0.74) [8,68]. Contrarily, several other studies have found the correlation between estimated and invasively measured pressure gradients to be weak (*r* = 0.35 to 0.47) [69,70,71], and the published results for estimating pressure gradient with Doppler US in aortic coarctation are diverse.

Oshinski et al. estimated pressure gradients from PC-MRI data and reported superior accuracy compared with estimated values derived from Doppler US, when compared with invasively measured pressure gradients [72]. No correlation coefficient between the methods was reported, and only a minority of the patients had invasive catheterization performed, i.e., 6 out of 32 patients. Pressure gradients have also been estimated from PC-MRI data using computational fluid dynamics simulations [73,74,75,76]. Simulated fluid motions based on Navier–Stokes equations have been reported to agree well with invasively measured pressure gradients before (*r* = 0.97, *p* = 0.00 and *r* = 0.85, *p* < 0.001) and after dilation of aortic coarctation (*r* = 0.87, *p* = 0.00) [75,76].

### 5.4. Coronary Arteries

Coronary CT angiography is the initial method for low-risk patients suspected of having coronary artery disease [77], thus replacing the conventional diagnostic coronary angiography with a non-invasive imaging modality. However, CT angiography is only used for mapping and characterizing anatomy and not for hemodynamic assessment.

Deng et al. published a study on PC-MRI data in flow phantoms for pressure estimation in the coronary arteries using the Navier–Stokes equations, and a strong correlation between estimated and invasively measured pressure gradients was reported (*r* = 0.97) [78]. When the technique was applied to patients, the pressure gradient was observed to increase exponentially with increasing stenosis severity. The paper did not report a correlation coefficient for the patient study. The limited amount of MRI-studies investigating the accuracy of pressure gradients in the coronary arteries, may be due to several obstacles: Viscous losses become more significant in small vessels, making the simplified Bernoulli (Equation (1)) equation less useful [79]. Furthermore, cardiac and respiratory motion, and the small size of the vessels, all affect the assessment of flow [78,80,81].

Likewise, only few studies have investigated the use of US for coronary arteries. Artifacts caused by calcifications, adjacency to the lung, cardiac, and respiratory motion, and the branching nature of the coronary arteries, all contribute to making it difficult to evaluate these vessels with US [82,83,84]. No studies investigating the use of Doppler US for estimating pressure gradients in the coronary arteries were found.

## 6. Estimation of Pressure Gradients in Carotids and Peripheral Vessels

Comparative studies between non-invasive estimation of pressure gradients and catheterization have also been performed in vessels outside the thoracic region. Doppler US, CT angiography, and MRI angiography are all recommended as diagnostic imaging methods for assessing the severity of peripheral artery disease [85]. Invasive angiography is only indicated, when non-invasive methods are inconclusive, or when revascularization is clinically indicated. Correlations were reported to be weak to moderate for the carotid arteries, when Illig et al. compared invasively measured pressure gradients with estimated pressure gradients derived from Doppler US using the simplified (*n* = 77, *r* = 0.419, *p* < 0.0001) (Equation (1)) and the expanded Bernoulli equation (*n* = 77, *r* = 0.374, *p* = 0.0008) (Equation (2)) [86]. Doppler US studies of the iliac arteries have reported results to be diverse with correlations ranging from weak to strong. In studies that applied the expanded Bernoulli equation (Equation (2)), the estimated pressure gradient was found to overestimate and to correlate from weakly to moderately with invasively measured values (*n* = 261, *r* = 0.27 and *n* = 33, *r* = 0.54) [87,88]. De Smet et al. suggested the low correlation to be caused by a large Doppler angle secondary to the course of the iliac arteries [87]. Langsfeld et al. and Strauss et al. used the simplified Bernoulli equation (Equation (1)) and reported the correlation between estimated and invasively measured pressure gradients to be strong, when performed on the iliac arteries (*n* = 23, *r* = 0.9, *p* < 0.01 and *n* = 28, *r* = 0.76, *p* < 0.0001) [89,90].

Several animal studies have evaluated the accuracy of non-invasive methods in peripheral vessels. Pressure gradients have been calculated by applying the Navier–Stokes equations to PC-MRI data in surgically created cerebral aneurysms [91], carotid stenoses [80,92] and renal artery stenoses [80,93] resulting in an overall strong correlation with invasive catheterization (*r* = 0.82 to 0.95).

The referenced papers in this section are listed in Table 1.

## 7. Discussion

Catheterization with pressure transducers is considered the gold standard for measuring intravascular pressures, but is an invasive procedure associated with risk of complications and ionizing radiation for guidance. Instead, US has been recommended for hemodynamic assessment of nearly all thoracic vessels, as the method is widely accessible, inexpensive compared with other methods, and provides real-time diagnostic images with an overall strong correlation with invasive measurements in the heart. When image quality of US is insufficient, or if the results do not agree with the clinical findings, MRI can be used as an alternative method to US [16,94]. Both US and MRI are desired methods as they are non-invasive and do not require ionizing radiation for imaging. 

Though, non-invasive estimation of pressure gradients is not without limitations. Estimated pressure gradients are calculated from blood flow velocities (Equations (1)–(3)) and are dependent on the velocity estimation to be precise. However, a gold standard for flow velocity estimation has not yet been established [31,32,33,34], making the evaluation of the accuracy of non-invasive velocity estimation difficult. Most of the studies comparing non-invasive estimation of pressure gradients with invasive catheterization used the simplified or expanded Bernoulli equation (Equations (1) and (2)) to convert a velocity into a pressure gradient. These equations neglect blood viscosity and pressure recovery, and the results may overestimate invasively measured pressure gradients [23,72,88,95,96]. Another source of error is that US produces images in two dimensions, but velocity estimation with Doppler US is only measured in one dimension, making it prone to error due to angle dependency [42,44]. MRI does not encounter this problem, as MRI measures flow velocities in three dimensions, but MRI is performed over multiple cardiac cycles with low temporal resolution, and the accuracy of velocity estimation may decrease further in vessels with large changes in flow velocity [50,97].

Additionally, comparison of non-invasive instantaneous pressure gradients with invasive pressure gradients measured with the peak-to-peak method poses another problem. Pressure gradients measured instantaneously and peak-to-peak will be different, as the pre-stenotic peak pressure does not occur simultaneously with the post-stenotic peak pressure during systole [7,90]. As the pre-stenotic pressure reaches its peak, the post-stenotic pressure will still increase [69,90], and thus the peak-to-peak pressure gradient is a non-physiological parameter [7]. The difference in pressure gradient between the peak-to-peak method and the instantaneous method was evaluated by Strauss et al. and Houston et al., who reported the mean differences to be 7 mmHg (*p* < 0.05; *r* = 0.76, *p* < 0.001) and 7.5 mmHg (2 SD: −45.4 to 30.3 mmHg), respectively [69,90]. Following these findings, studies comparing instantaneous non-invasive pressure gradients with peak-to-peak invasive measurements would be expected to report a positive systematic bias, if the non-invasive method is precise.

Doppler US is routinely used by cardiologists for estimation of blood pressure gradients, so its accuracy has already been widely accepted by many clinicians. Catheterization is still used, when non-invasive methods do not agree with the clinical findings [16]. However, Vecchi et al. reported catheters to overestimate pressure wires by 24% in vitro [9]. This finding was supported by computer simulations that found the overestimation to be 29%, while the catheter was present in the phantom. When the catheter was removed, the discrepancy in pressure measurement between pressure wire and the computer simulations was reduced to 1.5%. Therefore, using a catheter as a reference standard is questionable, as catheters narrow existing stenoses further and alters flow, thus overestimating the “actual” pressure gradient and disease severity [9,12,13]. Comparison of non-invasive methods with an inaccurate measuring method will at best result in it being just as inaccurate. Use of thinner pressure wires in clinical practice still remain less common due to technical complications, increased expenses, and the need for more extensive training of operators [9]. Most studies included in this review have used fluid-filled catheters to measure invasive pressures, and simultaneous invasive catheterization with non-invasive pressure gradient estimation was rarely performed. This resulted in the stenosis being less severe, when the non-invasive pressure gradient was being estimated.

Discrepancies in measurements can lead to undesired consequences. If the invasively measured pressure gradient is assumed to measure the true value and correctly reflect disease severity, then inaccurate estimation with non-invasive methods may result in misclassification of severity and can result in an individual being offered the wrong treatment strategy. Fisher et al. defined a pulmonary artery systolic pressure estimate by Doppler to be accurate if it was within 10 mmHg of the catheter measurement [64], but they found the Doppler US to be inaccurate in 48% of the cases (*n* = 65). Rich et al. reported similar findings, when they used the same criteria for accuracy and reported Doppler US to be inaccurate in 50.6% of cases (*n* = 160) [98]. However, despite the risk of misclassification, Doppler US is still recommended by multiple guidelines as the first-in-line method to diagnose vascular disease [16,45,47,85], as the availability and ease of use still outweighs the risks and costs of an invasive procedure. The use of non-invasive methods for hemodynamic evaluation in clinical work relies on its ability to detect a clinically significant obstruction and to correctly classify the severity. The daily use of Doppler US by many clinicians is proof that the accuracy of US is within an acceptable threshold, but is ultimately dependent on the clinical context.

Following the advancement in computer technology, more studies have investigated pressure gradients calculated with the Navier–Stokes equations (Equation (3)) using PC-MRI data. None of the studies in this review evaluating estimated pressure gradients derived with Doppler US have used the Navier–Stokes equations.

**Figure 3:**
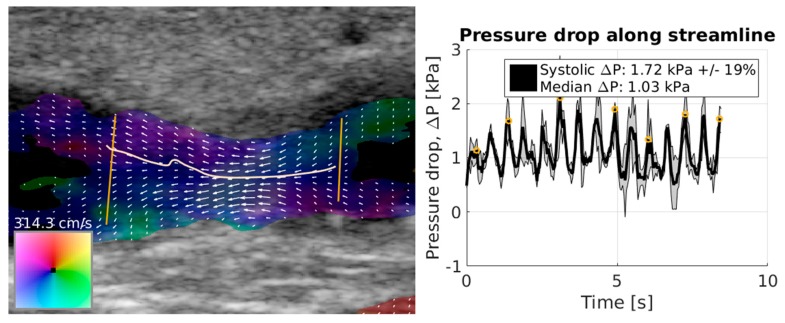
The **left** image shows vector velocity imaging of an arteriovenous fistula in a patient before intravascular balloon treatment of a stenosis. Arrows and colors represent the two-dimensional vector velocities as indicated by the color-box. Two vertical lines are manually placed, and a streamline representing the highest velocity between the two vertical lines is drawn. The **right** image shows the peak pressure gradient as a function of time along the plotted streamline over several heartbeats. Both the image and graph were created with an application provided by Jacob B. Olesen (BK Medical, Herlev, Denmark).

Olesen et al. suggested a method for estimation of pressure gradients based on the transverse oscillation method (Figure 3) [20,99]. The transverse oscillation method obtains angle-independent two-dimensional vector velocities, and by using either the Navier–Stokes equations (Equation (3)) or the non-steady Bernoulli equation, the authors estimated pressure gradients along plotted streamlines in healthy subjects [20,100]. Brandt et al. measured peak velocities with transverse oscillation and conventional Doppler US, and compared the estimates with MRI. Transverse oscillation was reported to be more accurate and precise than Doppler US for velocity estimation [101], which may translate into improved pressure gradient estimation.

Two-dimensional vector velocities may prove to be essential to understand the complex intravascular hemodynamics in circulation [40]. By visualizing and measuring flow in two dimensions rather than one, it may be possible to overcome some of the limitations experienced with conventional Doppler US, e.g., the angle dependency [40]. Unfortunately, out-of-plane motion in the third axis may still occur. Three-dimensional vector velocity estimation using US has been proposed and validated in vivo by Holbek et al. [102], but it has not yet been used for estimating pressure gradients. 

## 8. Conclusions

Overall, pressure gradients estimated with MRI and Doppler US correlate well with invasively measured pressure gradients. However, velocity estimation by Doppler US and calculation of pressure gradients using the simplified Bernoulli equation has several limitations, and future developments should aim for more advanced techniques. Using MRI, vector velocity US or possibly three-dimensional US in combination with the Navier–Stokes equations may provide a more accurate evaluation of flow hemodynamics, assisting the clinician in treatment of cardiovascular disease.

## Figures and Tables

**Table 1:** Shows a list of included studies in this review. “Simultaneous Doppler US” involved Doppler US to be performed simultaneously with catheterization. “Instantaneous gradients” is the correlation between a maximum Doppler US or MRI derived pressure gradient compared with an invasive pressure gradient measured using a dual-head catheter, which allowed for simultaneous invasive pressure measurement before and after a stenosis. “Peak-to-peak” is the correlation between maximum Doppler US or MRI derived pressure gradient compared with an invasive pressure gradient measured between the peak pressures before and after a stenosis. “Peak gradient” did not clearly specify how invasive pressure gradients were measured. “Mean gradient” is the correlation between a Doppler US or MRI derived mean pressure gradient compared with an invasively measured mean pressure gradient. * A correlation coefficient for the post-treatment comparison was not reported.

Study	Year	Country	Study Population (Subjects)	Methods Used	Results
Aortic and Mitral Valve					
Hatle et al. [17]	1978	Norway	Mitral valve stenosis (*n* = 35) Other valve lesions (*n* = 20)	Simultaneous Doppler US and simultaneous left and right heart catheterization. Doppler US before and after simultaneous left and right heart catheterization.	Reports good correlation between Doppler US and catheter gradients
Hegrenaes et al. [42]	1985	Norway	Aortic valve stenosis (*n* = 87)	Doppler US and catheter pullback	Reported underestimation by Doppler US
Stamm et al. [46]	1983	USA	Aortic valve stenosis (*n* = 26) Mitral valve stenosis (*n* = 27)	Doppler US and simultaneous left ventricular and femoral artery catheterization.	Aortic valve stenosis: *r* = 0.95 Mitral valve stenosis: *r* = 0.85
Knutsen et al. [51]	1982	Norway	Mitral valve stenosis (*n* = 16)	Simultaneous Doppler US and simultaneous left and right heart catheterization.	Mean gradient: *r* = 0.83 Mean difference: −4.9 mmHg
Hatle et al. [52]	1980	Norway	Aortic valve stenosis (*n* = 37)	Doppler US and catheterization	Reported underestimation by Doppler US in 8 out of 37 patients
Currie et al. [53]	1985	USA	Aortic valve stenosis (*n* = 100)	Simultaneous Doppler US and dualhead-catheter Simultaneous Doppler US and catheter pullback	Mean gradient: *r* = 0.92, mean difference: −4 mmHg Instantaneous gradient: *r* = 0.92, mean difference: −19 mmHg Peak-to-peak gradient: *r* = 0.91
Currie et al. [54]	1986	USA	Cardiac lesions (*n* = 95)	Simultaneous Doppler US and dualhead-catheter	Instantaneous gradient: *r* = 0.95, mean difference: −4 mmHg Peak-to-peak gradient: *r* = 0.92
Burstow et al. [55]	1989	USA	Prosthetic valves (*n* = 36)	Simultaneous Doppler US and dualhead-catheter	Mean gradient: *r* = 0.97 Instantaneous gradient: *r* = 0.94 Peak-to-peak gradient: *r* = 0.72, mean difference: −1 mmHg
Yeager et al. [56]	1986	USA	Aortic valve stenosis (*n* = 52)	Doppler US and catheter pullback	Mean gradient: *r* = 0.87 Peak-to-peak gradient: *r* = 0.84
Ohlsson et al. [57]	1986	Sweden	Aortic valve stenosis (*n* = 24)	Doppler US and simultaneous aortic arch and left ventricle catheterization	Mean gradient: *r* = 0.92 Instantaneous gradient: *r* = 0.89
Baumgartner et al. [58]	1999	Austria	Aortic valve stenosis (*n* = 21)	Doppler US and dualhead-catheter	Non-corrected instantaneous gradient: *r* = 0.95, mean difference: +18 mmHg Corrected instantaneous gradient: *r* = 0.97, mean difference: +0.4 mmHg Non-corrected mean gradient: *r* = 0.93, mean difference: +12 mmHg Corrected mean gradient: *r* = 0.96, mean difference: +1.1 mmHg
VanAuker et al. [59]	2000	USA	Aortic valve stenosis (*n* = 14)	Simultaneous Doppler US and dualhead-catheter	Mean difference: +42%
Yamazawa et al. [60]	2010	Japan	Aortic valve stenosis (*n* = 13)	Doppler US and catheter pullback	Non-corrected mean gradient: *r* = 0.98, mean difference: +5.7 mmHg Non-corrected peak-to-peak gradient: *r* = 0.87, mean difference: +8.5 mmHg Corrected mean gradient: *r* = 0.91, mean difference: −4.9 mmHg Corrected peak-to-peak gradient: *r* = 0.79, mean difference: −9.1 mmHg
Eichenberger et al. [61]	1993	Switzerland	Aortic valve stenosis (*n* = 19)	Doppler US (*n* = 15) MRI (*n* = 19) Catheterization (*n* = 13)	Doppler US vs catheter: *r* = 0.96 MRI vs catheter: *r* = 0.97
Pulmonary Hypertension					
Hioka et al. [63]	2017	Japan	Patients referred for right heart catheterization (*n* = 55)	Doppler US and catheterization	Tricuspid gradient: *r* = 0.73, mean difference: +2.52 mmHg
Fisher et al. [64]	2009	USA	Pulmonary hypertension (*n* = 65)	Doppler US and catheterization	Pulmonary artery systolic pressure: *r* = 0.66, mean difference: −0.6 mmHg Tricuspid mean difference: −1.8 mmHg
Janda et al. [66]	2011	Canada	Pulmonary hypertension	Meta-analysis	Pulmonary artery systolic pressure: *r* = 0.70 Summary sensitivity: 83% Summary specificity: 72%
Nogami et al. [67]	2009	Japan	Pulmonary hypertension (*n* = 20)	Doppler US, MRI and catheterization	Pulmonary artery systolic pressure: Doppler US vs. catheter: *r* = 0.86 MRI vs. catheter: *r* = 0.94
Coarctation of Aorta					
Syamasundar et al. [8]	1989	Saudi Arabia	Coarctation of Aorta (*n* = 28)	Doppler US and catheter pullback	Simple Bernoulli: *r* = 0.76 Expanded Bernoulli: *r* = 0.76
Marx et al. [68]	1986	USA	Coarctation of aorta (*n* = 28)	Doppler US and catheter pullback	Simple Bernoulli: *r* = 0.91, mean difference: +8 mmHg Expanded Bernoulli: *r* = 0.98, mean difference: 0 mmHg
Houston et al. [69]	1987	Scotland	Coarctation of aorta (*n* = 46)	Doppler US, catheter pullback, dualhead-catheter, two catheters	Instantaneous gradient: *r* = 0.36 Peak-to-peak: *r* = 0.42
Wisotzkey et al. [70]	2015	USA	Aortic arch obstruction (*n* = 60)	Doppler US and catheter pullback	Simple Bernoulli: *r* = 0.47 Expanded Bernoulli: *r* = 0.35
Tang et al. [71]	2009	USA	Coarctation of aorta (*n* = 34)	Doppler US and catheter pullback	Peak-to-peak gradient: *r* = 0.37, mean gradient: *r* = 0.001 Reported overestimation of peak pressure gradient by Doppler US
Oshinski et al. [72]	1996	USA	Coarctation of aorta (*n* = 32)	Doppler US (*n* = 22) MRI (*n* = 22) Catheterization (*n* = 6)	Reported a non-significant difference between MRI and catheter gradients after correction after a new suggested model
Itu et al. [73]	2013	USA	Coarctation of aorta (*n* = 4)	Computer simulation on patient MRI data and catheterization data	Reported good agreement between MRI and catheter gradients
Sotelo et al. [74]	2015	France	Coarctation of aorta (*n* = 7)	Simultaneous MRI and catheterization.	Reported good agreement between MRI and catheter gradients.
Goubergrits et al. [75]	2015	Germany	Coarctation of aorta (*n* = 13)	MRI and simultaneous ascending aorta and femoral catheterization	Peak gradient: Pre-treatment: *r* = 0.97, Mean difference: −0.5 mmHg (*p = 0.8*) Post-treatment: *r* = 0.87, Mean difference: +3.0 mmHg (*p* = 0.00)
Mirzaee et al. [76]	2017	Germany	Coarctation of aorta (*n* = 12)	MRI and catheterization	Pre-treatment: *r* = 0.85, Mean difference: −0.58 mmHg (*p = 0.64*) Post-treatment: *r* = NA*, Mean difference: −2.54 mmHg (*p = 0.04*)
Coronary Artery					
Deng et al. [78]	2017	USA	Coronary artery stenosis (*n* = 6)	MRI and catheterization	Reported a trend between MRI and catheterization
Carotid Artery					
Illig et al. [86]	1996	USA	Carotid artery stenosis (*n* = 76)	Doppler US, and direct puncture of the common carotid and internal carotid artery	Simplified Bernoulli: *r* = 0.374 Expanded Bernoulli: *r* = 0.419
Iliac Artery					
De Smet et al. [87]	2000	Netherlands	Iliac artery stenosis (*n* = 261)	Doppler US and dual-catheter before and after treatment.	Instantaneous gradient: *r* = 0.27 Reported overestimation by Doppler US
Kohler et al. [88]	1987	USA	Iliac artery stenosis (*n* = 18)	Doppler US and catheterization	Expanded Bernoulli: *r* =0.54
Langsfeld et al. [89]	1988	USA	Iliac artery stenosis (*n* = 11)	Doppler US and catheter pullback	Pressure gradient: *r* = 0.9
Strauss et al. [90]	1993	Germany	Iliac artery stenosis (*n* = 28)	Doppler US and catheter pullback	Mean gradient: *r* = 0.77, mean difference: −2 mmHg Instantaneous gradient: *r* = 0.80, mean difference: −1 mmHg Peak-to-peak gradient: *r* = 0.76, mean difference: −7 mmHg
Animal studies					
Lum et al. [80]	2007	USA	Porcine with surgically created stenosis (*n* = 12)	MRI and dual-catheter in: Carotid stenosis (*n* = 12) Renal artery stenosis (*n* = 9) Iliac stenosis (*n* = 9)	Mean gradients: Carotid artery: *r* = 0.891 Renal artery: *r* = −0.0815 Iliac artery: *r* = 0.915 Mean difference for carotid + iliac arteries: +0.86 mmHg
Moftakhar et al. [91]	2007	USA	Canines with surgically created carotid bifurcation aneurysm (*n* = 8)	MRI and catheterization	Intra-aneurysmal pressures: *r* = 0.82
Turk et al. [92]	2007	USA	Canines with surgically created carotid bifurcation stenosis (*n* = 6)	MRI and catheter pullback	Pressure gradient: *r* = 0.86
Bley et al. [93]	2011	USA	Porcine with surgically created renal artery stenosis (*n* = 12)	MRI and two catheters before and after stenosis	Peak gradient: *r* = 0.91 Mean gradient: *r* = 0.98
